# Naturally acquired antibodies to gametocyte antigens are associated with reduced transmission of *Plasmodium vivax* gametocytes to *Anopheles arabiensis* mosquitoes

**DOI:** 10.3389/fcimb.2022.1106369

**Published:** 2023-01-16

**Authors:** Surafel K. Tebeje, Wakweya Chali, Elifaged Hailemeskel, Jordache Ramjith, Abrham Gashaw, Temesgen Ashine, Desalegn Nebret, Endashaw Esayas, Tadele Emiru, Tizita Tsegaye, Karina Teelen, Kjerstin Lanke, Eizo Takashima, Takafumi Tsuboi, Nichole D. Salinas, Niraj H. Tolia, David Narum, Chris Drakeley, Benoit Witkowski, Amelie Vantaux, Matthijs M. Jore, William J. R. Stone, Ivo S. Hansen, Fitsum G. Tadesse, Teun Bousema

**Affiliations:** ^1^ Armauer Hansen Research Institute, Malaria and Neglected Tropical Disease Directorate, Addis Ababa, Ethiopia; ^2^ Department of Medical Microbiology, Radboud University Medical Center, Nijmegen, Netherlands; ^3^ Department of Biology, College of Natural and Computational Sciences, Wollo University, Dessie, Ethiopia; ^4^ Division of Malaria Research, Proteo-Science Center, Ehime University, Matsuyama, Ehime, Japan; ^5^ Laboratory of Malaria Immunology and Vaccinology, Division of Intramural Research, National Institute of Allergy and Infectious Disease, National Institutes of Health, Bethesda, MD, United States; ^6^ London School of Hygiene & Tropical Medicine, London, United Kingdom; ^7^ Malaria Molecular Epidemiology Unit, Pasteur Institute of Cambodia, Phnom Penh, Cambodia

**Keywords:** *Plasmodium vivax*, gametocyte, transmission, immunity, anopheles, malaria, transmission-blocking, vaccine

## Abstract

Naturally acquired antibodies may reduce the transmission of *Plasmodium* gametocytes to mosquitoes. Here, we investigated associations between antibody prevalence and *P. vivax* infectivity to mosquitoes. A total of 368 microscopy confirmed *P. vivax* symptomatic patients were passively recruited from health centers in Ethiopia and supplemented with 56 observations from asymptomatic *P. vivax* parasite carriers. Direct membrane feeding assays (DMFA) were performed to assess mosquito infectivity; for selected feeds these experiments were also performed after replacing autologous plasma with malaria naïve control serum (n=61). The prevalence of antibodies against 6 sexual stage antigens (Pvs47, Pvs48/45, Pvs230, PvsHAP2, Pvs25 and PvCelTOS) and an array of asexual antigens was determined by ELISA and multiplexed bead-based assays. Gametocyte (ρ< 0.42; p = 0.0001) and parasite (ρ = 0.21; p = 0.0001) densities were positively associated with mosquito infection rates. Antibodies against Pvs47, Pvs230 and Pvs25 were associated with 23 and 34% reductions in mosquito infection rates (p<0.0001), respectively. Individuals who showed evidence of transmission blockade in serum-replacement DMFAs (n=8) were significantly more likely to have PvsHAP2 or Pvs47 antibodies. Further studies may demonstrate causality for the observed associations, improve our understanding of the natural transmission of *P. vivax* and support vaccine development.

## 1 Introduction

The significant reduction in malaria burden in the first decade of this millennium has plateaued since 2015 and even reverted in some settings in recent years (WHO, 2015). Current intervention strategies appear insufficient to achieve malaria elimination in the majority of endemic countries (Griffin et al., 2010). Whilst most malaria-attributed deaths are due to *Plasmodium falciparum*, *Plasmodium vivax* also has a high clinical burden with an estimated 4.5 million cases in 2020 (WHO, 2015). *P. vivax* appears particularly hard to eliminate (Price et al., 2020), principally due to its ability to form dormant liver stages that can result in relapsing infections unless treated with an effective radical cure. Another challenge in controlling and eliminating *P. vivax* infections is the rapid formation of gametocytes, the parasite life stage responsible for onward transmission to mosquitoes. Once taken up by blood-feeding *Anopheles* mosquitoes, gametocytes activate to become male and female gametes that fuse to form a motile ookinete. This ookinete penetrates the mosquito gut to develop into an oocyst that releases sporozoites that ultimately render the mosquito infectious upon its next bite. Whilst gametocyte maturation is a long process in *P. falciparum*, infectious *P. vivax* gametocytes appear in the bloodstream within 48 hours of blood stage infection (Bousema and Drakeley, 2011). The strong association between asexual parasite and gametocyte density (Koepfli et al., 2015) make clinical *P. vivax* malaria cases highly infectious to mosquitoes at the moment of clinical presentation (Martins-Campos et al., 2018; Tadesse et al., 2018). Given that *P. vivax* parasites are capable of infecting a wide variety of *Anopheles* mosquitoes (Rios-Velásquez et al., 2013; Araújo et al., 2020), it is unsurprising that *P. vivax* transmission is highly efficient in many settings.

Naturally acquired human immune responses can reduce or fully prevent the transmission of *Plasmodium* parasites from humans to mosquitoes (recently reviewed in (de Jong et al., 2020)). For *P. vivax*, the first empirical evidence for naturally acquired transmission blocking activity (TBA) (Koepfli et al., 2015) came from studies conducted in the 1980s and 1990s in Sri Lanka (Mendis et al., 1987; Peiris et al., 1988; Gamage-Mendis et al., 1992). In these studies, investigators fed mosquitoes with blood from acutely infected Sri Lankan individuals. In paired feeding experiments, gametocyte infectivity was compared between a condition where the patient’s autologous plasma was present in the bloodmeal versus a condition where this plasma was replaced with malaria naïve control serum. In most of these paired experiments, transmission was reduced in the presence of autologous plasma (indicative of plasma derived TBA) although in some experiments the autologous plasma had the opposite effect and thus enhanced transmission (Peiris et al., 1988; Gamage-Mendis et al., 1992). In supporting experiments, indirect immunofluorescence tests showed that sera with TBA were more reactive to gamete surface antigens compared to sera without this functional activity. Moreover, purified immunoglobulins from these suppressive sera blocked transmission when added to a gametocytaemic bloodmeal, confirming this TBA to be antibody-mediated (Peiris et al., 1988). Subsequent studies in Mexico (Ramsey et al., 1994), and Colombia (Arévalo-Herrera et al., 2005) similarly demonstrated the existence of naturally acquired *P. vivax* TBA. However, the antibody specificity or strength of the associations remain largely undescribed. Several *P. vivax* gametocyte antigens have been developed as transmission blocking vaccine candidates. Antibody responses that are elicited by these proteins can reduce *P. vivax* transmission, as demonstrated by ookinete culture experiments or by mosquito feeding experiments where serum from vaccinated animals was added to gametocytes from naturally infected malaria-positive blood donors and offered to mosquitoes. These experiments have confirmed the potency of several pre-fertilization antigens including Pvs48/45 (Tachibana et al., 2015; Arévalo-Herrera et al., 2022), Pvs230 (Tachibana et al., 2012), Pvs47 (Tachibana et al., 2015) and PvHAP2 (Qiu et al., 2020) and post-fertilization antigens Pvs25 and Pvs28 (Hisaeda et al., 2000). Lastly, *P. vivax* Cell-traversal protein for ookinetes and sporozoites (PvCelTOS) (Kariu et al., 2006; Jimah et al., 2016; Kumar et al., 2022) was identified as potential inducer of TBA (Mehrizi et al., 2017). Recognition of these recombinant proteins has so far not been systematically studied in malaria-endemic populations or related to *P. vivax* transmission efficiency.

Here, we therefore investigated the relationship between the infectivity of *P. vivax* infections to locally reared *An. arabiensis* mosquitoes and naturally acquired antibodies against *P. vivax* gametocyte antigens in symptomatic and asymptomatic infections in Ethiopia.

## 2 Methods

### 2.1 Study design and population

This study was conducted in Adama district and Metehara town of Oromia Regional State and Arba Minch in Southern Region, Ethiopia. Adama district is characterized by low and seasonal malaria transmission that primarily occurs during the long (July to September) and short (May to June) rainy seasons (Golassa and White, 2017). Metehara town has low perennial malaria transmission with peaks in incidence from September to November and March to May. Arba Minch is characterized by moderately intense perennial malaria transmission. *P. falciparum* and *P. vivax* are co-endemic in all study sites where *Anopheles arabiensis* is a primary malaria vector (Tekeste et al., 2013; Tadesse et al., 2018; Abossie et al., 2020).

Across sites, microscopy confirmed symptomatic *P. vivax* patients were recruited at health centers from December 2017 to March 2022 (**Table 1**). Demographic data was collected from patients or their guardians (if minors) at the moment of presentation. Patients were included in the study if they had microscopy-confirmed *P. vivax* infections, had symptoms suggestive of malaria (defined as axillary temperature ≥37.5°C or history of fever, alone or in combination with nausea, chills and headaches) and were ≥2 years of age. Patients were excluded if they had a chronic and/or an acute illness that required immediate clinical care or bleeding disorders. Hemoglobin concentration was measured by HemoCue photometer (HemoCue 201+, Angelholm, Sweden).

**Table 1:** Characteristics of study participants recruited between December 2017 and March 2022.

Characteristics	Symptomatic	Asymptomatic
Age, median (IQR)	19 (13 - 28)	14 (9 - 21.5)
Asexual parasites/µL (microscopy), median (IQR)	4752.0 (1547.0 - 10706.0)	0.0 (0.0 - 0.0)
Gametocyte prevalence, microscopy (% (n/N))	36.4 (134/368)	0.0 (0.0/56)
Gametocyte prevalence (*Pvs25*), RT-qPCR (% (n/N))	76.1 (280/368)	80.4 (45/56)
Mosquito infectious individuals, % (n/N)	73.9 (272/368)	0.0 (0.0/56)*
Infected mosquitoes, % (n/N)	37.0 (4036/10,879)	0.0 (0.0/1,800)

*24 asymptomatic patients with 56 longitudinal data points were included.

Symptomatic (n= 368); Asymptomatic (n=24, with 56 longitudinal mosquito feeding moments); Total (n= 424). Abbreviations: IQR, inter quartile range.

All data points selected for the asymptomatic individuals were *P. vivax *positive by RT-qPCR.

Observations from clinical malaria cases were complemented with observations from asymptomatic infections. These comprised samples from qPCR confirmed *P. vivax* infections collected during the same study period from asymptomatic individuals who were enrolled in a longitudinal observational study on the dynamics of asymptomatic infections in Adama district (Hailemeskel, Tebeje et al. in preparation). Samples from these individuals were included in the current analysis to ensure coverage of a broad range of transmissible and non-transmissible gametocyte densities to allow more precise fitting of the associations between parasite and gametocyte densities and mosquito infection rates. For asymptomatic parasite carriers, afebrile community members were eligible if they were above the age of 2 years and positive for *P. vivax* parasites by qPCR, without symptoms suggestive of malaria in the past 48 hours and with no evidence of chronic and/or acute illness. Venous blood samples were collected every 14 days for 2-months and used in mosquito feeding assays and for plasma collection. For the current study, we randomly selected samples from this cohort without the intention to describe (longitudinal) patterns in parasite carriage but to obtain an informative population of low parasite and gametocyte densities for curve-fitting.

### 2.2 Ethics statement

All participants or their guardians (if minor) provided written informed consents to participate in the study. The study protocol was approved by the Ethics Review Committees of the Armauer Hansen Research Institute (AHRI) (protocol number: PO32/18 and PO35/17), the Ethics committee of the London School of Hygiene and Tropical Medicine (LSHTM Ethics ref: 15811), and the National Research Ethics Review Committee (SHE/S.M./14.4/708/19) at the Ministry of Science and Higher Education of The Federal Democratic Republic of Ethiopia.

### 2.3 Study procedures

#### 2.3.1 Blood sampling

Venous blood was collected from each participant for mosquito feeding, molecular assays and serology. Three mL of blood was collected in either EDTA (BD K2E EDTA Vacutainers) or serum tubes (BD Vacutainer Plus Serum) for nucleic acid collection and plasma/serum collection. An additional 2mL was collected in Heparin tubes (BD Lithium Heparin Vacutainers) for mosquito feeding experiments (to measure mosquito infectivity) since other anticoagulants interfere with transmission efficiency (Graumans et al., 2022). Plasma (1:1 in 0.05% NaAz) and serum were stored at -70°C for serological investigations.

#### 2.3.2 Parasite quantification

Asexual parasites and gametocytes were detected and quantified by microscopy (WHO, 2010). For quantification of gametocytes with reverse transcriptase quantitative PCR (RT-qPCR), total nucleic acids were extracted from 100µL whole blood stored in 500µL RNAProtect (Qiagen) and extracted by MagNAPure LC automatic extractor (Roche applied Sciences). RT-qPCR targeted *Pvs25* mRNA, an established marker of female gametocytes (Wampfler et al., 2013; Tadesse et al., 2018). All primer and probe sequences and combinations are included in supplementary file (**Table S3**).

#### 2.3.3 Infectivity assessment

Mosquito membrane feeding assays were performed as described previously (Ouédraogo et al., 2013; Tadesse et al., 2018) to assess infectivity to locally reared *An. arabiensis* mosquitoes. Briefly, a heparinized whole blood sample was fed to ~ 40 (4 - 7 days old) female *An. arabiensis* mosquitoes per cup (three cups with a total of 120 mosquitoes per experiment) using water jacketed glass feeders (0.3mL capacity) maintained on a water bath to keep the temperature in the feeders at 37°C. Mosquitoes were starved for ~12 hours prior to feeding. For the serum replacement mosquito feedings, heparinized whole blood (~833µL) was centrifuged at 1800 × g for 5 minutes in a pre-warmed (37°C) centrifuge to collect autologous plasma (~500µL). Pre-warmed (37°C) ~500µL naïve AB European serum (Sanquin, Nijmegen, the Netherlands) was used to replace the autologous plasma. Fully fed mosquitoes were maintained on 10% sucrose solution (g/mL) at 26℃ ± 2 room temperature and 60% ± 10 humidity post feeding. Mosquitoes (30 per experiment) were dissected on day 7 post feeding, and midguts were stained with 1% mercurochrome for microscopic detection and quantification of oocysts.

#### 2.3.4 Serological analyses

Antibody levels against *P. vivax* antigens, Pvs25, Pvs48/45, Pvs47, Pvs230 and PvCelTOS were assessed by Enzyme Linked Immunosorbent Assay (ELISA). Pvs25, Pvs48/45, Pvs47 and Pvs230 were expressed using a wheat germ cell-free (WGCF) system (CellFree Sciences, Matsuyama, Japan) (Liu et al., 2022); an additional Pvs230 was expressed in *Pichia pastoris* (Pvs230D1M (Tentokam et al., 2019)); and the sporozoite/ookinete antigen PvCelTOS was expressed in *E. coli* (Jimah et al., 2016; Jimah et al., 2017a; Jimah et al., 2017b; Kumar et al., 2022). ELISA plates were coated with 1µg/mL (in PBS) of antigen overnight at 4°C. After thorough washing with home-made PBS-Tween (0.05%), plates were blocked with 5% skimmed milk in 0.05% PBS-Tween for 1 hour at room temperature (RT). Plates were then washed. Diluted test samples/controls in 1% skimmed milk (in 0.05% PBS-Tween) were added to the plates and incubated for 2 hours at RT. After a washing step, Goat anti-human IgG-HRP (1:50,000) (Pierce 31412) in PBS-Tween (0.05%) was added and plates were incubated at RT for 2 hours. Following a final washing step, a colorizing substrate TMB (K-Blue substrate, Neogen, Sigma), was added and incubated for ~20 minutes. After ~20 minutes of incubation, a stopping solution of 0.2M Sulphuric acid (H_2_SO_4,_Merck KGaA cat 100731) was added to stop the reaction. Plates were immediately read by plate reader (Bio-Rad, iMark microplate reader) at 450 nm. OD values were used as measure of antibody density; antibody prevalence was assessed by a mixture model, taking the mean plus two standard deviations from the negative population as cut-off for positivity (Wu et al., 2020). A more conservative approach with three standard deviations is sometimes used (Stewart et al., 2009) but more appropriate for larger sample sizes. The availability of WGCF proteins was a limiting factor and not all samples could be processed by ELISA; samples with serum replacement observations were prioritized for a complete dataset.

Alongside ELISAs, we tested a panel of 7 P*. vivax* antigens from different life stages in a bead based multiplex immunoassay for comparison; one pre-erythrocytic stage (Circumsporozoite protein [VK210 CSP]), 5 asexual blood stage (Merozoite surface protein 1-19 [MSP1-19], Apical membrane antigen 1 [AMA1], Duffy binding protein [DBP RII], Reticulocyte binding protein [RBP 2b], and Erythrocyte binding protein [EBPII]), and one sexual stage (HAP2). Antibody responses were quantified using a Luminex MAGPIX^©^ suspension bead array, as described previously (Wu et al., 2019). Briefly, plasma/serum samples were assayed at a dilution of 1:400. Secondary antibody was an R-phycoerythrin conjugated goat anti-human IgG (Jackson Immuno Research, PA, USA; 109-116-098) diluted to 1:200. Data are presented as background adjusted median fluorescence intensities (MFI).

### 2.4 Data analyses

Statistical analyses were performed using STATA (version 14.2, StataCorp., TX, USA) and R (version 4.1.1). Proportions were compared using McNemar’s test for paired observations, and Pearson χ2 test or Fisher exact test for independent observations. Spearman rank correlation coefficient (ρ) was used to assess associations between continuous variables. Continuous variables were presented as medians and interquartile ranges (IQRs). TBA was calculated as percent inhibition in oocyst density in naïve sera feeds compared to whole blood feeds (Lensen et al., 1996). A cut-off for antibody positivity was determined by first using a mixture model to distinguish two Gaussian distributions. The cut-off was determined as the estimated 97.5th percentile of the underlying Gaussian distribution for the negative antibodies (i.e. 2 standard deviations above the mean of the negative population). Logistic and Linear mixed effects regression models were used for multivariate analyses. Analysis with p<0.05 were considered statistically significant.

## 3 Results

### 3.1 Participant characteristics

**Figure 1:**
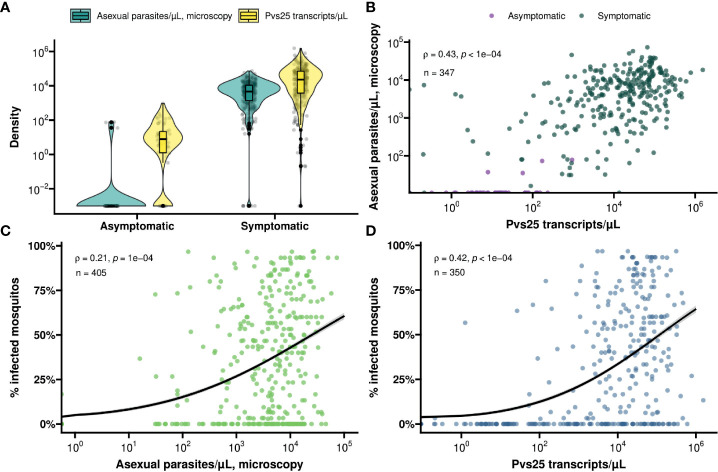
**(A)** Violin and box plot for the distribution of molecularly determined gametocyte density (yellow) and microscopically detected asexual parasite density (turquoise) by symptom status. **(B)** A scatter plot for the association between asexual parasite density and gametocyte density by symptom status (asymptomatic – violet, symptomatic – green). Spearman’s ρ = 0.43 indicating a moderate positive monotonic association (p<0.0001). **(C)** Scatter plot showing the association between asexual parasite density and mosquito infectivity. Spearman’s ρ = 0.21 indicating a weak positive monotonic association (p<0.0001). The black line shows the fit from a logistic regression model and the grey shading shows the 95% confidence interval. **(D)** Scatter plot showing the association between gametocyte density and mosquito infectivity. Spearman’s ρ = 0.42 indicating a moderative positive monotonic association (p<0.0001). The black line shows the fit from a logistic regression model and the grey shading shows the 95% confidence interval.

In total, 368 symptomatic patients with a median age of 19 years (interquartile range (IQR), 13 – 28 years) were enrolled in the study between December 2017 and March 2022 (**Table 1**). Of these participants 47.8% (174/364) had an active fever and 0.6% (2/332) had hemoglobin levels <8g/dL. In these patients, the median asexual parasite density by microscopy was 4752.0 parasites/µL (IQR, 1547.0 - 10706.0) and 36.4% (134/368) had microscopically detected gametocytes. By Pvs25 RT-qPCR, *P. vivax* gametocytes were detected in 76.1% (280/368) of samples with median *Pvs25* transcript numbers of 22245.9 copies/µL (IQR, 3770.4 - 70980.8). These data from clinical malaria patients, who typically harbor high parasite and gametocyte densities (McKenzie et al., 2006; Pukrittayakamee et al., 2008), were supplemented with a total of 56 follow-up observations from 24 asymptomatic parasite carriers who were for the most part microscopy negative for malaria parasites but qPCR positive for *P. vivax* and thus carried very low density infections (Nguyen et al., 2018) (**Table 1**). Asymptomatic parasite carriers had a median age of 14 years (IQR, 9 - 21.5). Microscopically quantified asexual parasites and molecularly quantified gametocyte densities differed markedly between symptomatic and asymptomatic populations (**Figure 1A**). When combining observations from both symptomatic and asymptomatic populations, gametocyte density was positively associated with parasite density (**Figure 1B**, ρ = 0.43; p < 0.0001).

### 3.2 Mosquito infection rates are associated with parasite and gametocyte density.

**Figure 2:**
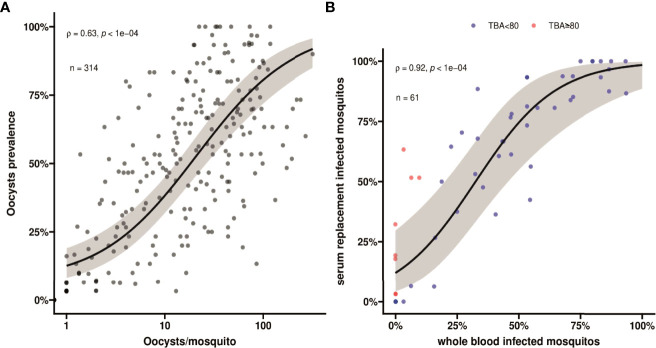
**(A)** A scatter plot showing association between oocyst prevalence, or mosquito infection rates, and mean oocyst per mosquito. Spearman’s ρ =0.63 indicating a moderate positive monotonic association p<0.0001. The black line shows the fit from a logistic regression model and the grey shading shows the 95% confidence interval. **(B)** A scatter plot indicating the association in mosquito infection rates between whole blood and serum replacement feeds. Observations with TBA<80% are indicated in blue and TBA≥80% are indicated in red. Spearman’s ρ = 0.92 indicates a strong positive monotonic association (p<0.0001). The black line shows the fit from a logistic regression model and the grey shading shows the 95% confidence interval. The plot also indicates that infectivity in the serum replacement condition (median 60.6%, IQR 6.3% - 85.2%) was consistently higher than in the whole blood condition (median 33.3%; IQR 0% - 57.6%) (p<0.0001). This value is different, but not contradicting, the comparison in the main text where we calculated the pairwise differences and report the median and IQR for the pairwise differences.

For all clinical patients and asymptomatic parasite carriers, whole blood was offered to locally reared *An. arabiensis* mosquitoes that were examined 7 days later for infection status. For 61 clinical patients recruited between March 2020 and March 2022, these mosquito feeding experiments were also conducted after replacing autologous plasma with malaria-naïve serum. Asexual parasite density was positively associated with the proportion of mosquitoes that became infected when feeding on whole blood of parasite carriers (**Figure 1C**); this association was stronger for molecularly quantified gametocyte density (**Figure 1D**). Oocyst prevalence, or the proportion of infected mosquitoes, was strongly positively associated with mean oocyst density (ρ = 0.63; p < 0.0001) (**Figure 2A**).

Despite these statistically significant associations, many individuals with relatively high gametocyte densities did not infect mosquitoes. For the subset of participants whose blood was used in serum replacement experiments (n=61), we observed a strong positive association in mosquito infection rates between the whole blood and serum replacement feeds (**Figure 2B**; ρ = 0.92, p<0.0001). We observed higher mosquito infection rates for the control serum condition with a median increase of 13.2% (IQR, 0% - 25.0%) infected mosquitoes. We expressed the lower mosquito infection rates in the autologous plasma condition relative to that in the naïve serum condition as transmission blocking activity (TBA). This TBA ranged from -80% (enhancement) to 100% (complete blockade). For those showing higher infection rates in whole blood conditions (n=5), this ‘transmission enhancement’ was mostly very weak (3.2 - 12.1%). Only 1 individual showed more than 80% enhancement with the proportion of infected mosquitoes being 15.6% for whole blood and 6.3% for serum replacement conditions. In contrast, 13.1% (8/61) of experiments showed greatly increased infection rates following serum replacement (>80% higher infection rates and thus TBA >80%). TBA estimates for five experiments were not interpretable since infection rates were zero in the whole blood and serum replacement conditions.

### 3.3 Transmission efficiency is associated with gametocyte immune responses

**Figure 3:**
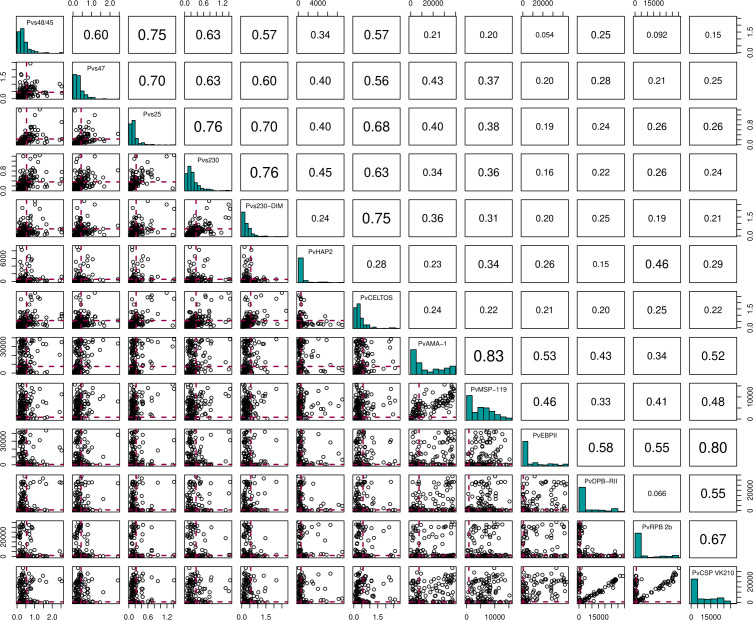
Correlation matrix plot for the 13 different antigens. Here the diagonal shows the histogram distribution for each of the antigens. The triangle above the diagonal shows the Spearman's Correlation Coefficient for every pair of antigens on the diagonal. The bottom triangle shows the scatter plots for every pair of antigens on the diagonal. Here, the red dashed lines indicate the cut-offs for antibody positivity.

To test a possible impact of anti-gametocyte immune responses on transmission efficiency, we determined antibody responses to sexual stage antigens Pvs47, Pvs48/45, Pvs230 (two variants), PvCelTOS and Pvs25 by ELISA. These assessments were done for 224 individuals, including all those participating in serum replacement mosquito feeding experiments. Due to antigen scarcity, not more samples were completed by ELISA. Additional antigens, including sexual-stage antigen PvHAP2, were measured by bead-based multiplex assay. Antibody positivity was defined based on a mixture model, assuming an antibody positive and antibody negative population for each antigen within the study population. The optical density values in ELISA, reflective of antibody densities, were strongly correlated between most of the antigens (**Figure 3**. For instance, responses to the two Pvs230 variants were strongly correlated (ρ = 0.68, p<0.0001), as were responses to Pvs230 and Pvs25 (ρ = 0.63, p<0.0001). Among individuals with microscopy-positive infections, antibody prevalence was weakly associated with a lower parasite density at the time of sampling for PvDPB-RII (p = 0.06) but not for any of the other antigens (**Table S1**)

**Figure 4:**
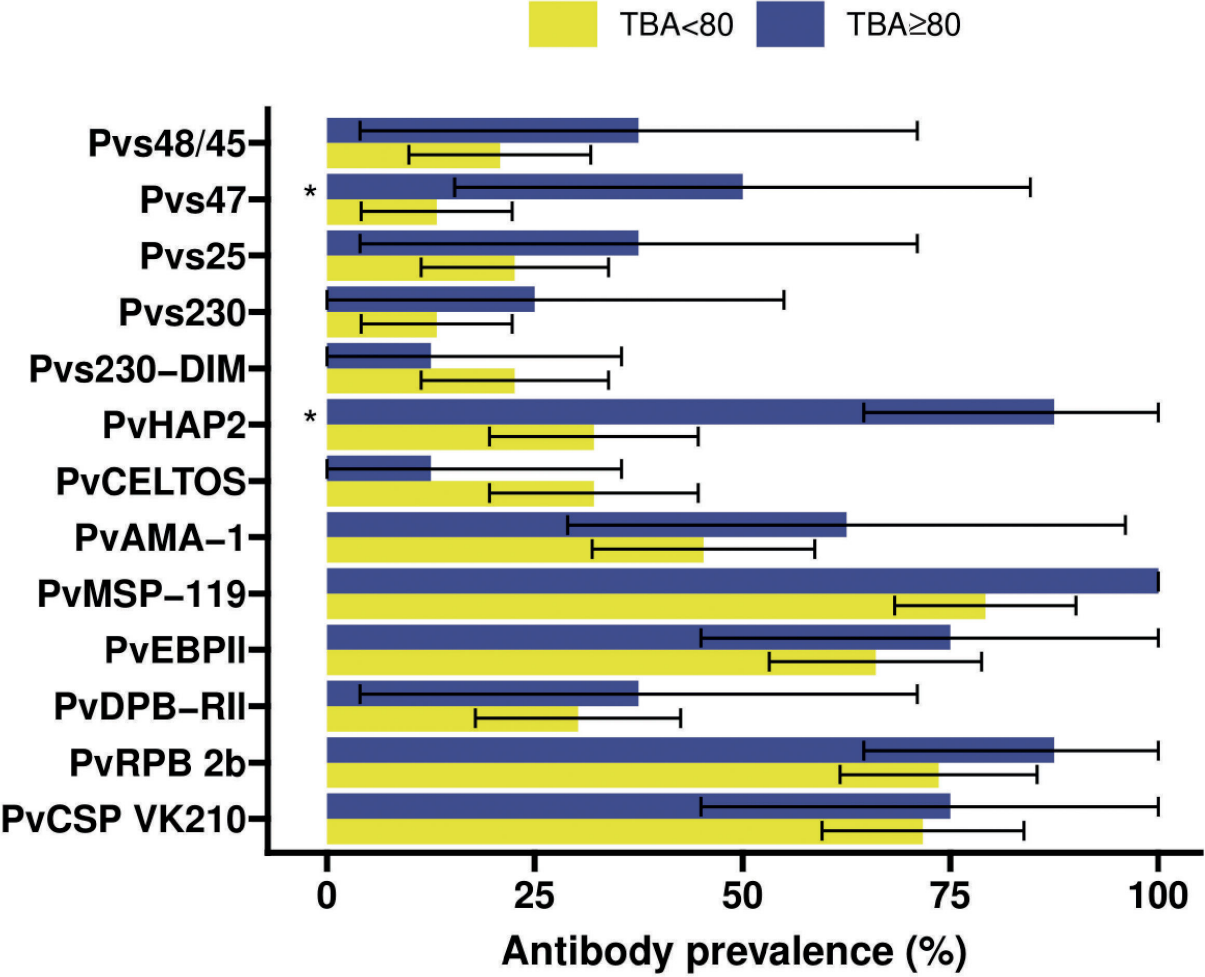
Antibody prevalence (Kim et al., 2011) for the 13 different antigens stratified for whether the transmission blocking activity (TBA) was at least 80% (blue) or less (yellow). 95% confidence intervals are shown by the black bars. Significant (p<0.05) differences in prevalence between TBA<80% and TBA≥80% are indicated by * where p-values were calculated from Fisher’s exact tests.

**Figure 5:**
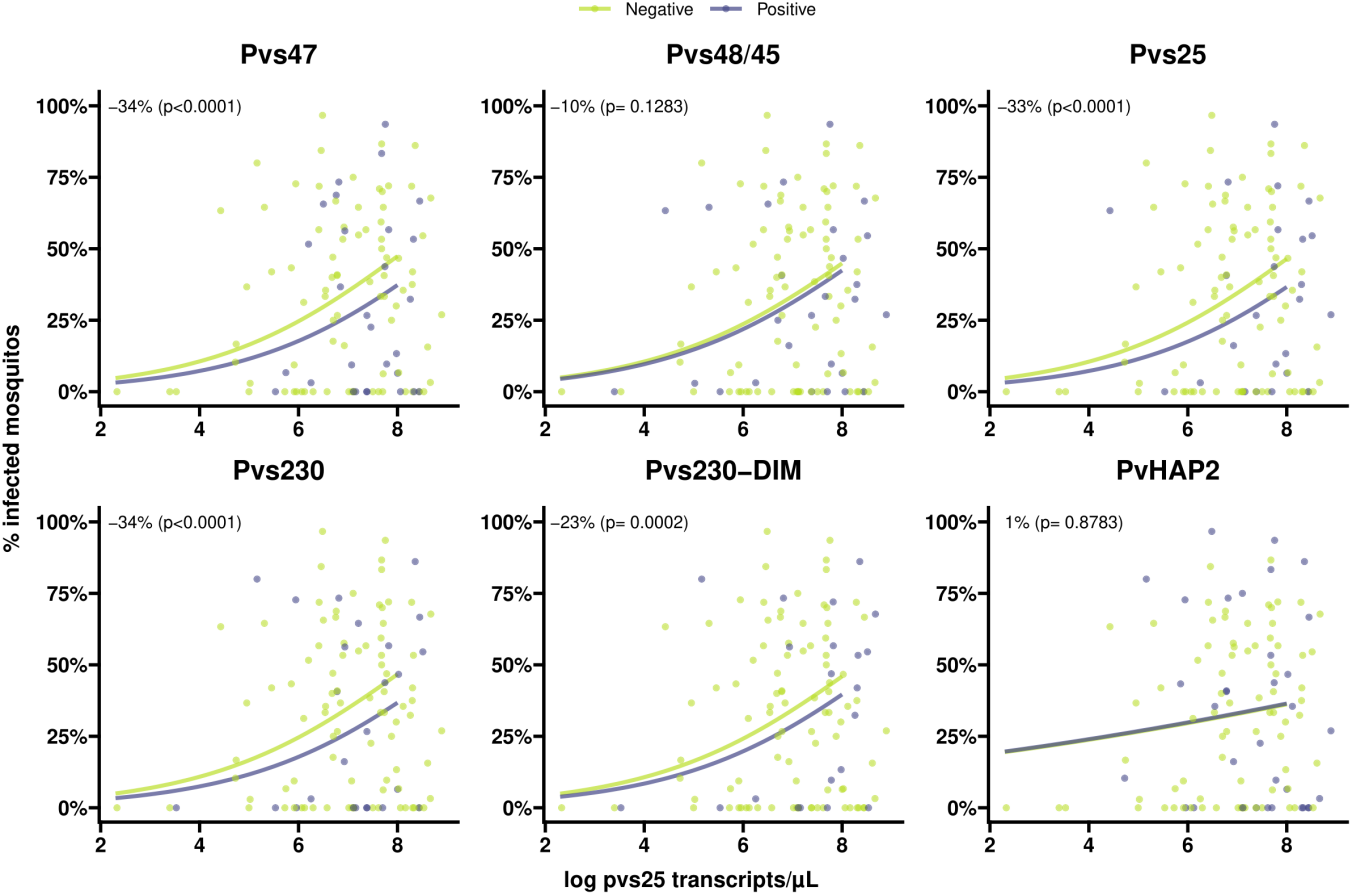
Scatter plots indicating the association between gametocyte density and antibody prevalence on mosquito infectivity for six different antigens. Positive antibodies are indicated in light blue and negative antibodies are indicated in green. The light blue and green lines shows the fit from logistic regression models for the association between gametocyte density and mosquito infectivity for positive and negative antibodies respectively. The numbers in the plot (estimated from logistic regression models) indicate the average difference in mosquito infectivity between positive and negative antibodies while accounting for gametocyte density, and the p-values indicate whether or not these differences are significantly different from 0%.

Individuals with >80% TBA were more likely to be antibody positive for most gametocyte antigens (**Figure 4**), although this difference only reached statistical significance for Pvs47 and PvHAP2 (**Table S2**). We did not observe any evidence of associations with >80% TBA for any of the asexual antigens (**Figure 4**, **Table S2**). We next examined whether antibody prevalence was also associated with transmission efficiency in the larger dataset where whole blood samples were offered to mosquitoes and gametocytes were quantified by molecular methods. We thus determined whether the proportion infected mosquitoes for a given gametocyte density was lower in the presence of gametocyte antibodies. The presence of antibodies against Pvs47 was associated with a relative reduction in mosquito infection rates of 34% (p<0.0001). Similarly, Pvs230 and Pvs25 antibody prevalence were associated with statistically significant relative reductions of 23 and 34%, respectively; for Pvs48/45 antibodies a smaller and non-significant reduction was observed (**Figure 5**). For PvCelTOS and asexual stage antigens, highly heterogeneous effects were observed. The presence of antibodies against CSP and RBP-2b was associated with 61% and 20% increases in mosquito infection rates, respectively; while antibodies against AMA-1 were associated with a 26% decrease in mosquito infection rates (p<0.0001) (**Figure S1**).

## 4 Discussion

Here, we described the association of antibody responses to a portfolio of gametocyte and non-gametocyte antigens with *P. vivax* transmission efficiency. We observed higher prevalences of antibodies against gametocyte and ookinete antigens Pvs47, Pvs48/45, Pvs230, PvHAP2 and Pvs25 among individuals with high levels of transmission blocking activity with statistically significant differences for Pvs47 and PvHAP2. No such association was found for antibodies against PvCelTOS and to a panel of asexual and sporozoite antigens. Antibody responses against Pv47 and Pvs25 were further associated with a lower per-gametocyte infectivity when whole blood samples were offered to mosquitoes.

The transmission of *P. vivax* parasites to mosquitoes is strongly associated with gametocyte density (Kiattibutr et al., 2017; Tadesse et al., 2018; Vantaux et al., 2018) that is in turn associated with asexual parasite density (Wampfler et al., 2013; Tadesse et al., 2018; Vantaux et al., 2018). Whilst the positive association between mosquito infection rates and gametocyte density is also observed for *P. falciparum* (Da et al., 2015; Bradley et al., 2018), for *P. vivax* this association is particularly strong and very high mosquito infection rates and infection intensities are commonly observed (Kiattibutr et al., 2017; Tadesse et al., 2018; Vantaux et al., 2018). At present, *Pvs25* is the most widely used mRNA target to quantify circulating gametocytes although it may not accurately reflect gametocyte maturity or infectivity. These high mosquito infection rates and limited inter-person variation in per-gametocyte infectivity, suggest that host immune factors may play a relatively minor role in dictating transmission efficiency. Nevertheless, anti-gametocyte antibody responses have been observed for *P. vivax* and some of the most compelling evidence for immune-modulatory properties of serum factors comes from *P. vivax* infections (Mendis et al., 1987; Peiris et al., 1988; Gamage-Mendis et al., 1992) where antibodies may both reduce and enhance transmission (reviewed in (Stone et al., 2019)). In our cohort of patients with clinical *P. vivax* infections, 13.1% (8/61) individuals reduced transmission by more than 80%. We classified these individuals as individuals having high TBA. This arbitrary cut-off is commonly used in malaria transmission research although it is acknowledged that also lower levels of TBA may have a meaningful impact on transmission (Blagborough et al., 2013). Too few individuals (n=5) enhanced transmission and the magnitude of transmission enhancement was very modest for most individuals. We concluded that this did not allow for meaningful analyses of immune responses associated with enhancement in our cohort. Also our number of transmission-blockers (n=8) was very modest but the effect was much larger with 80-100% reduction in the proportion of infected mosquitoes in whole blood compared to serum replacement conditions.We observed that the prevalence of antibodies against gametocyte antigens Pvs47 (OR=6.57; 95% CI: 1.33 – 32.48, p=0.0209) and PvHAP2 (OR=14.82; 95% CI: 1.69 – 130.25, p=0.015) were significantly higher among individuals with this strong TBA (>80%) compared to those with lower levels of TBA. Whilst the small number of observations warn that these findings have to be interpreted with caution, it is striking that none of the asexual antigens was associated with TBA>80%, suggesting that cumulative prior exposure to *P. vivax* may not be predictive of functional transmission-reducing immunity.

We detected antibodies against the ookinete protein Pvs25 in ~25% of individuals which is in line with a previous study (Kim et al., 2011) where 19.2% of naturally infected patients had detectable Pvs25 antibodies. *P. falciparum* Pfs25 mRNA is translationally repressed (Lasonder et al., 2016) and no or very low levels of antibodies are detected in naturally exposed individuals (Miura et al., 2013; Stone et al., 2018; Essangui et al., 2019). In rodent models, Pys25, an ortholog of Pvs25 and Pfs25 in *P. yoelii*, protein expression was detected in gametocytes (Tachibana et al., 2018). Our findings suggest that translational repression could be incomplete (leaky expression) in *P. vivax* similar as in *P. yoelii* (Tachibana et al., 2018). Strikingly, the presence of Pvs25 antibodies was associated with lower transmission-efficiency in our broader dataset. Similar associations were observed for Pvs47, where antibody prevalence was associated with an estimated 34% reduction in mosquito infection rates, and Pvs230. PvCelTOS can be expressed on surfaces of both sporozoites and ookinetes (Mehrizi et al., 2017), and we speculated that antibodies against PvCelTOS expressed on sporozoites could influence ookinete development, and thus transmission. However, in our cohort, we observed no association between PvCelTOS antibodies and either TBA or general transmission efficiency.

Our study has several limitations. First of all, our findings were primarily based on clinical malaria cases who are highly infectious in *P. vivax* (Kiattibutr et al., 2017; Vantaux et al., 2018) and were therefore considered an informative population to examine changes in transmission efficiency. However, asymptomatic infections may also be relevant for the human infectious reservoir in some *P. vivax* endemic settings (Tadesse et al., 2018) and will have a different infection history and therefore plausibly a different antibody profile. Our findings can thus not be extrapolated to asymptomatic populations. We also used two platforms to quantify antibody responses. After initial attempts to couple antigens to beads for Luminex assays which were unsuccessful for all antigens except for HAP2, we used ELISA for all antigens other than HAP2. Most importantly, we describe epidemiological associations and our findings do not provide conclusive evidence for a causal role of anti-gametocyte antibodies in dictating *P. vivax* gametocyte infectivity. Whilst our findings for several antigens with an established role in gametocyte fertilization are biologically plausible and supported by pre-clinical studies where vaccine-induced antibodies were causally associated with TRA and also broadly corroborate findings in *P. falciparum*, causality would require a different set of experiments. For *P. falciparum*, antibodies specific for Pfs48/45 and Pfs230 were purified from large-volume plasma samples and offered to mosquitoes in the presence of cultured gametocytes (Stone et al., 2018). Similar experiments would allow demonstrating causality for antibodies against *P. vivax* gametocyte antigens but would have to rely on natural gametocyte donors since continuous culture of *P. vivax* has not been established. In addition, we did not collect the volumes of plasma (up to 9mL) that was used to demonstrate the functionality of antibodies against Pfs48/45 and Pfs230 in *P. falciparum* (Stone et al., 2018). Furthermore, our study did not test these associations for different concentrations of antibodies or dilutions of autologous plasma. We also did not examine TBA after depleting plasma of antibodies using the recombinant proteins; this would have allowed us to explore TBA associated with antibodies to antigens other than the panel examined here or antibody-independent TBA. It is therefore important to interpret the described associations with caution, also demonstrated by positive and negative associations of mosquito infection rates with antibodies against several asexual antigens.

In conclusion, our mosquito feeding experiments on naturally infected Ethiopians provide evidence for a plausible role of antibodies against *P. vivax* sexual stage antigens in determining the transmission efficiency of *P. vivax* gametocytes to locally relevant *An. arabiensis* mosquitoes. These findings, that require confirmation in other populations, can help understand the natural transmission of *P. vivax* and support the development of transmission-blocking vaccine candidates.

## Data availability statement

The raw data supporting the conclusions of this article will be made available by the authors, without undue reservation.

## Author contributions

Conceived, designed and supervised the study: TB, FGT. Wrote manuscript: SKT, TB, FGT. Edited manuscript: CD, BW, AV, MMJ, WJRS, ISH, WC, EH, NS, NT, DN. Performed experiments: SKT, WC, EH, AG, TA, NDS, EE, TE, TseT, KT, KL, ISH, WJRS. Analysed data: JR, TB, SKT. Provided validated antigens: ET, TsuT, NS, NT. All authors contributed to the article and approved the submitted version. 
