# Supplementary material for: Naturally acquired antibodies to gametocyte antigens are associated with reduced transmission of Plasmodium vivax gametocytes to Anopheles arabiensis mosquitoes
Source: Front Cell Infect Microbiol. 2023 Jan 16;12:1106369. doi: 10.3389/fcimb.2022.1106369 (PMC9885094; doi:10.3389/fcimb.2022.1106369)
Supplement: Supplementary file 1 [file DataSheet_1.docx]

| **Table S1. Parasite density ratio between antibody positive and negative samples** | | | | | | |
| --- | --- | --- | --- | --- | --- | --- |
| **Antigens** | **Density ratio** | **Lower CI** | **Upper CI** | **p-value** |  |  |
| Pvs47 | 1.03376551 | 0.555177543 | 1.92491779 | 0.9167441 |  |  |
| Pvs48/45 | 0.970252518 | 0.495599809 | 1.899496191 | 0.9298983 |  |  |
| Pvs25 | 0.595658352 | 0.282660158 | 1.255248973 | 0.1749864 |  |  |
| Pvs230 | 0.775207773 | 0.410002812 | 1.465714559 | 0.4344639 |  |  |
| Pvs230-DIM | 0.852875156 | 0.432412687 | 1.682180133 | 0.646683 |  |  |
| PvCELTOS | 1.0036775 | 0.523660802 | 1.923704275 | 0.9911899 |  |  |
| PvAMA-1 | 0.890743838 | 0.423333609 | 1.874230081 | 0.761311 |  |  |
| PvEBPII | 0.816628842 | 0.388470056 | 1.716690015 | 0.5946083 |  |  |
| PvHAP2 | 1.08067882 | 0.487100072 | 2.397590925 | 0.8491537 |  |  |
| PvMSP-119 | 1.072617334 | 0.45735181 | 2.515586293 | 0.8723655 |  |  |
| PvDPB-RII | 0.488550347 | 0.236505021 | 1.009202429 | 0.0566313 |  |  |
| PvRPB 2b | 1.423115233 | 0.658281188 | 3.076583387 | 0.3725003 |  |  |
| PvCSP VK210 | 0.737979624 | 0.34484648 | 1.579293855 | 0.4361784 |  |  |

**Table S2. TBA of blocker and non-blocker sera**

| **Antibody** | **Blocker (TBA>80%)** | **Non-blocker** | **OR (95% CI), p** |
| --- | --- | --- | --- |
| Pvs47 | 50.0 (4/8) | 13.2 (7/53) | 6.57 (1.33, 32.48) , p=0.0209 |
| Pvs48/45 | 37.5 (3/8) | 20.8 (11/53) | 2.29 (0.47, 11.1) , p=0.3031 |
| Pvs25 | 37.5 (3/8) | 22.6 (12/53) | 2.05 (0.43, 9.85) , p=0.3700 |
| Pvs230 | 25.0 (2/8) | 13.2 (7/53) | 2.19 (0.37, 13.08) , p=0.3898 |
| Pvs230-DIM | 12.5 (1/8) | 22.6 (12/53) | 0.49 (0.05, 4.37) , p=0.5213 |
| PvCELTOS | 12.5 (1/8) | 32.1 (17/53) | 0.3 (0.03, 2.66) , p=0.2809 |
| PvHAP2 | 87.5 (7/8) | 32.1 (17/53) | 14.82 (1.69, 130.25) , p=0.015 |
| PvAMA-1 | 62.5 (5/8) | 45.3 (24/53) | 2.01 (0.44, 9.3) , p=0.3699 |
| PvEBPII | 75.0 (6/8) | 66.0 (35/53) | 1.54 (0.28, 8.43) , p=0.6168 |
| PvMSP-119 | 100.0 (8/8) | 79.2 (42/53) | 8103773.78 (0, Infty) , p=0.9894 |
| PvDPB-RII | 37.5 (3/8) | 30.2 (16/53) | 1.39 (0.3, 6.52) , p=0.6782 |
| PvRPB 2b | 87.5 (7/8) | 73.6 (39/53) | 2.51 (0.28, 22.28) , p=0.408 |
| PvCSP VK210 | 75.0 (6/8) | 71.7 (38/53) | 1.18 (0.21, 6.54) , p=0.8462 |

| **Table S3: Primers and Probes combinations** | |  |
| --- | --- | --- |
| **Primer type** | ***P. falciparum (18S)*** | ***P. vivax* (*Pvs25*)** |
| Forward | 5’-GTAATTGGAATGATAGGAATTTACAAGGT-3’ | 5'-ACA CTT GTG TGC TTG ATG TAT GTC-3' |
| Reverse | 5’-TCAACTACGAACGTTTTAACTGCAAC-3’ | 5'-ACT TTG CCA ATA GCA CAT GAG CAA-3' |
| probe | 5'-6FAM-AACAATTGGAGGGCAAG–MGBNFQ-3' | 5'-FAM-TGC ATT GTT GAG TAC CTC TCG GAA-BHQ1-3' |


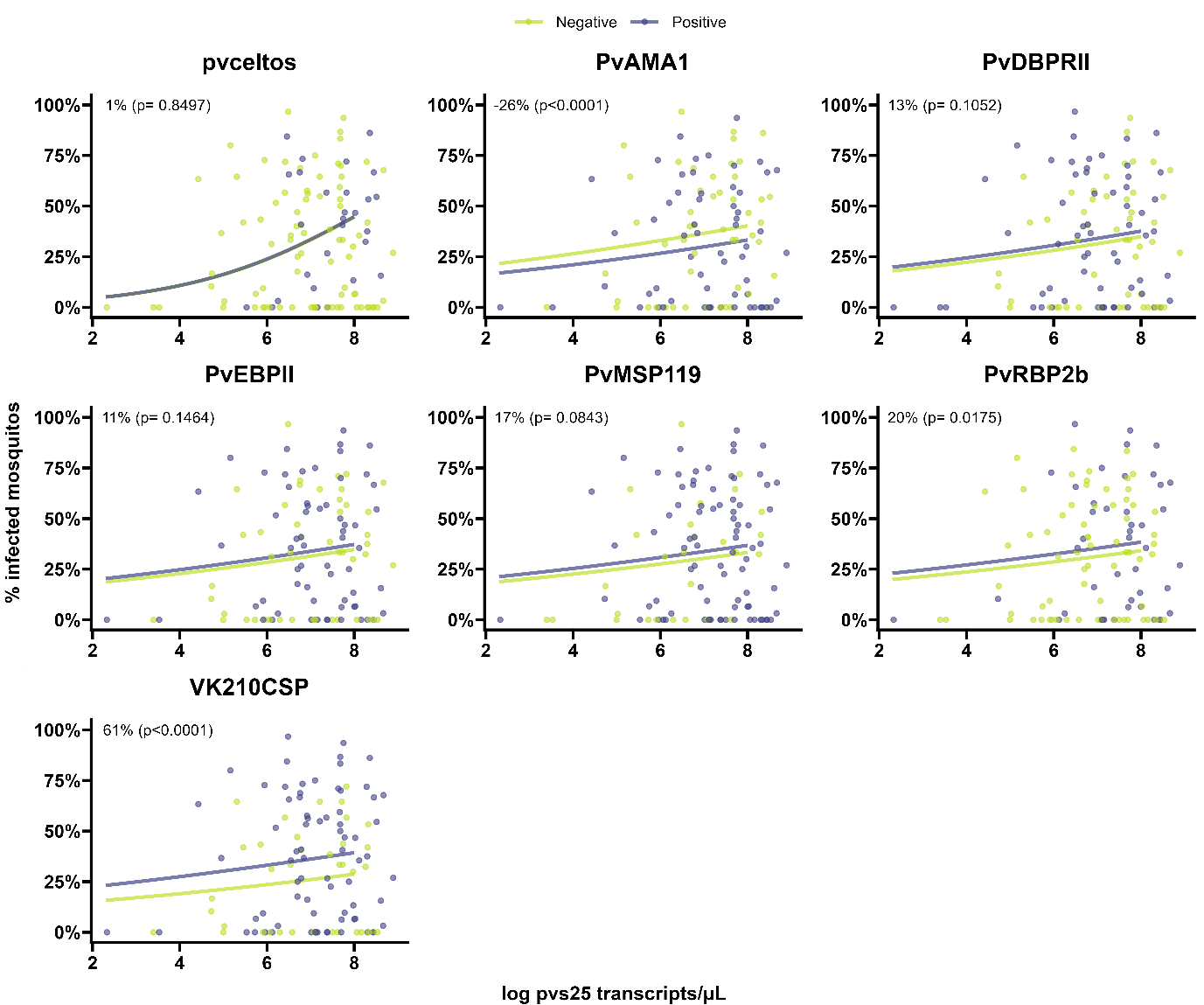


**Figure S1:** Scatter plots indicating the association between gametocyte density and antibody prevalence on mosquito infectivity for seven asexual stage antigens. Positive antibodies are indicated in light blue and negative antibodies are indicated in green. The light blue and green lines show the fit from logistic regression models for the association between gametocyte density and mosquito infectivity for positive and negative antibodies respectively. The numbers in the plot (estimated from logistic regression models) indicate the average difference in mosquito infectivity between positive and negative antibodies while accounting for gametocyte density and the p-values indicate whether or not these differences are significantly different from 0%.
